# Effectiveness of the Thermal Treatments Used for Curd Stretching in the Inactivation of Shiga Toxin-Producing O157 and O26* Escherichia coli*

**DOI:** 10.1155/2017/1609836

**Published:** 2017-08-22

**Authors:** M. Trevisani, A. Valero, R. Mancusi

**Affiliations:** ^1^Department of Veterinary Medical Sciences, University of Bologna, Via Tolara di Sopra 50, 40064 Ozzano dell'Emilia, Italy; ^2^Department of Food Science and Technology, University of Cordoba, International Campus of Excellence in the AgriFood Sector (ceiA3), Campus de Rabanales, Edificio Darwin, 14014 Córdoba, Spain

## Abstract

The kneading treatment of the fresh curd in hot water is a critical control point in the manufacturing of mozzarella. Factors such as the ratio between hot water and curd mass, the rheological properties, and the mixing and kneading activity affect the processing time and the internal temperature of the curd. The aim of this study was to investigate the effect of thermal treatments on the fate of Shiga toxin-producing* Escherichia coli* (STEC). Nine curd samples (weight 160–270 g) were artificially contaminated with O157 or O26 STEC and stretched in hot water (90–95°C) for 5–10 min. Depending on the heating process and spinning, different nonisothermal profiles were recorded. Observed reductions of O157 and O26 STEC varied between 1.01 and more than 5.38 log⁡MPN (Most Probable Number)/g at the end of the temperature treatments. Further, nonisothermal log-linear tail models were developed to compare observed reductions for O157 and O26 VTEC under variable temperature conditions. Results obtained showed that the comparison of predictions provided by the dynamic model with observations described well the linear inactivation pattern since nonsignificant differences were denoted at all profiles tested. The dynamic model developed can be useful to evaluate the effectiveness of the thermal treatments used in the manufacturing of mozzarella in the inactivation of STEC.

## 1. Introduction

Mozzarella is a high moisture soft cheese that belongs to the category of stretched curd cheeses. The use of fresh raw milk is indicated in the technical guideline of the “mozzarella di bufala campana PDO” (DMPAF, 2003), but the use of thermized or pasteurized milk is allowed. After a curd-ripening phase (4–4.5 h at 35–37°C), which occurs under whey, curd is cut in slices and put on tables for draining. The mature curd (pH of 4.9–5.1) is stretched into hot water (90–95°C) reaching temperatures of 58–65°C and the product is finally moulded to obtain a round shape [1]. The temperature treatment used to stretch the curd essentially pasteurizes this cheese [2–5], but the duration of the treatment is controlled empirically by cheese-makers based on the curd melting and stretching properties.

The epidemiological data show that the public health risk is low. Only two outbreaks were recently associated with cheese in Europe in 2013 and none involved mozzarella [6]. However, in 2005 three cases of Haemolytic Uremic Syndrome associated with an infection by* Escherichia coli* O26 were reported in Italy and the possible source was traced to mozzarella produced in a processing plant in the province of Salerno [7]. More recently, O26 STEC isolates were detected in two samples of raw milk and in one sample of mozzarella that was collected in Apulia region after an outbreak with 22 cases of HUS due to O26 STEC. The indistinguishable PFGE profile detected in the raw milk and mozzarella samples may be indicative of the persistence of the strain through the mozzarella cheese production process [8]. Some studies showed that Shiga toxin-producing* E. coli* (STEC) are present in buffalo's cow farms [9, 10]. The number of STEC in raw milk is probably very low, but studies aiming at assessing the growth of STEC during the milk fermentation, renneting, cutting, and cheddaring of curd are lacking. International regulatory agencies require that HTST pasteurization of milk is designed to ensure at least a 5-log reduction of* Mycobacterium tuberculosis*,* Mycobacterium bovis*, and* Coxiella burnetii* in whole milk (4% milkfat), for example, Codex Alimentarius [11] (2004) and PMO (2011) [12]. Advice to accomplish at least a 5-log cycle reduction was indicated by the US Food and Drug Administration for milk nontreated fruit juice [13], and 5D was used as a reference also for ground beef and acidified foods [14, 15].

A precautionary approach to the risk management would be to have at least 5-log reduction for STEC during the stretching phase.

A 5-log reduction of* E. coli* O157:H7 was observed after stretching curd made with cow milk in hot water at 80°C for 5 minutes by Spano et al. [4]. Fusco et al. [16] reported that although hot water (90°C) was used for stretching curd, the temperature in the core was around 55°C and after 5–8 min a reduction of 1.96 log⁡MPN (Most Probable Number)/g of* E. coli* O157:H7 was observed. When “pasta filata” is made, temperature dynamics cannot be easily controlled and the core of products (i.e., the inner part of the round shaped final product) does not correspond to the inner part during the handling made for stretching and spinning the curd in the hot water. A recent study concerning the effect of thermal treatments on Shiga toxin-producing* E. coli* in buffalo curd reports that the average time needed for a 4-log (4-D) reduction of strains of serovars O26 and O157 at a temperature range of 67 to 80°C was 2.1 to 2.6 min and 2.1 to 6.3 min, respectively [17]. However, that study was performed at static conditions using a convention heat transfer mechanism (i.e., tubes hold in a hotplate), which could not accurately reproduce the convection heat transfer that occurs when the curd is mixed with hot water.

In the present study, the effect of different thermal treatments on the fate of Shiga toxin-producing* E. coli* (STEC) was assessed at nonisothermal conditions and the kinetic inactivation parameters obtained at static conditions [17] were used to develop nonisothermal inactivation models. The fate of STEC observed in different challenge tests was compared with the rate of microbial population reduction estimated by the nonisothermal models.

## 2. Material and Methods

### 2.1. Strains and Culture Conditions

The strains used for the challenge tests corresponded to* E. coli* O157:H7 (stx1+, stx2+, eae+, Hly+, not fermenting sorbitol) and* E. coli* O26:H11 (stx1+, eae+, not fermenting rhamnose) that were isolated in 2013 from an in-line milk filter in a buffalo farm (strain O157) and from bulk cow's milk intended for pasteurization (strain O26), respectively [18]. Precultures were prepared for each individual batch experiment from the same cryovials (O157/O26) stored at −80°C by streaking on Trypticase Soy Agar plates (TSA). After 15–18 hours of incubation at 37°C, one colony was picked and inoculated in buffered peptone water (BPW) and cultures were incubated at 37°C for 18–24 hours. The transmittance (560 nm) of cultures was standardized at 40% to have a concentration of approximately 10^8^ CFU/ml and this was used for spiking the curd samples used for the challenge tests. The number of bacteria in the standardized BPW cultures was counted on TSA plates (after incubation at 37°C for 24 hours).

### 2.2. Samples

Nine loaves of curd (slices cut to be about 15 centimetres thick) for individual batch experiments were taken from a local artisanal cheese plant producing mozzarella. The curd loaves were brought into the laboratory in an insulated box within 5 minutes from collection and kept at room temperature until the pH was between 5.1 and 5.3. Curd subsamples (25 g portions) were taken to detect the presence of contaminating STEC. During the challenge test period (after the first two tests) the producer decided to modify the fermentative process and milk starter cultures of* Streptococcus thermophilus* were inoculated in lukewarm milk instead of the whey resulting from the previous day fermentation vat (known as “*cizza*”).

### 2.3. Challenge Test Conditions

Portions of the mature curd were cut into small fragments with a sterile blade and weighted (200–300 g) in polyethylene bags. 4 ml of a dilution of the standardized STEC cultures containing approximately 10^8^ CFU/ml was poured on the curd and the bags containing the contaminated curd were manipulated for approximately 15 min to break the curd into pieces and ensure a proper mixing and an even distribution of the inoculum. Twenty-five-gram aliquots were taken from each bag to assess the numbers of STEC in the curd samples before the heat treatments. For making “*pasta filata*” preheated boiling water (400 ml) was put into a ceramic vessel that was placed on an electric hot plate to maintain the temperature high (90–95°C). The weighted curd samples (range 160–270 g) were put in the hot water. The thermostatic temperature control of the hot plate was set to keep the water temperature close to 90°C during the challenge tests. The curd samples were spun and stretched using 20 cm long plastic pliers and Teflon spoon. A temperature probe (with a copper-plated sensor tip) was fastened to one arm of the pliers and wire-connected to a data logger. The curd was stretched for 5–10 min. (depending on the temperature conditions) until the rheological properties of “*pasta filata*” (stretchability) and appearance (smoothness, shininess, and even surface) were appropriate. Then, “pasta filata” was extracted from the water, round-shaped, and immediately chilled in ice water. The final product “mozzarella” was then analysed to assess the number of surviving STEC. Samples of water used for the thermal treatments were also taken at the end of the stretching step.

### 2.4. Microbiological Analyses of Curd Samples and Water

Twenty-five-gram curd subsamples were taken from the curd leaves and analysed with the standard method ISO/TS 13136:2012 [19] to detect the presence of Shiga toxin-producing* E. coli*. An enumeration method was used to quantitatively detect O157/O26* E. coli* in the inoculated curd samples taken before the heat treatments. To do this, 100 *μ*l of dilutions 1 : 10, 1 : 100, and 1 : 1000 of the sample homogenates (25 g in 225 ml BPW) was spread in duplicate on specific selective/differential media, Cefixime-Tellurite Sorbitol MacConkey Agar (CT-SMAC, Oxoid, UK) for STEC O157 and Cefixime-Tellurite Rhamnose MacConkey Agar (CT-RMAC, Lab M, Bury, UK) for STEC O26. The typical colourless colonies grown on CT-SMAC/CT-RMAC (O157 not fermenting sorbitol or O26 not fermenting rhamnose) were counted in the plates where their number was between 15 and 300. Five colonies were picked from these plates in order to confirm the serogroup using slide agglutination with* E. coli* O157 and O26 Latex test kits (Oxoid, UK).

The Most Probable Number method [20] was used instead for estimating the number of viable STEC in the pasta filata and hot water, because the low number of heat stressed surviving STEC can be easily detected by using a nonselective enrichment step, which was made in buffered peptone water (BPW). The real-time PCR (RT-PCR) method described by Perelle et al. [21] was used to detect the target bacteria (O157 or O26) in the cultures (MPN tubes) and 100 *μ*l of the cultures taken from the PCR positive tubes was streaked on SMAC and RMAC agar plates to confirm their viability. For this purpose, 25 g of curd and samples was homogenized in 225 ml of buffered peptone water (BPW). Then 10 ml of homogenates and subsequent serial dilutions (1 : 10, 1 : 100, and 1 : 1000 in BPW) in triplicate was incubated at 37°C for 24 h. Water samples were analysed taking 10, 1, and 0.1 ml of enriched water using appropriate volumes of BPW in stomacher bags and tubes (90 ml for the 10 ml subsamples and 9 ml for the 1 and 0.1 ml subsamples). Each subsample was analysed in triplicate to estimate the MPN of STEC. The bacterial growth in the BPW tubes/bags was evaluated and the presence of O157/O26 STEC was tested by RT-PCR and in the PCR positive tubes/bags by detection of colonies grown on SMAC and RMAC showing the typical morphology (O157, sorbitol negative on SMAC, and O26, rhamnose negative on RMAC). The latex agglutination tests were used to confirm the serogroup.

### 2.5. Records of Temperature Measurements

The temperature was measured during the challenge tests using a certified temperature data logger (Hobo® Pro series H08-031-08, Onset, USA) connected with a temperature sensor (TMC-20HD, Onset, USA) having a response time of 30 sec. in stirred water. Records of the temperature collected per second were stored in the data logger and exported into Excel™ to obtain time-temperature graphs. The probe was fixed with nylon cable ties to a plastic rod that was used along with a spoon to stretch the curd for obtaining the “pasta filata,” which is then moulded manually to produce one piece of mozzarella of approximately 150–200 g that was immediately cooled in ice water.

**Figure 1:**
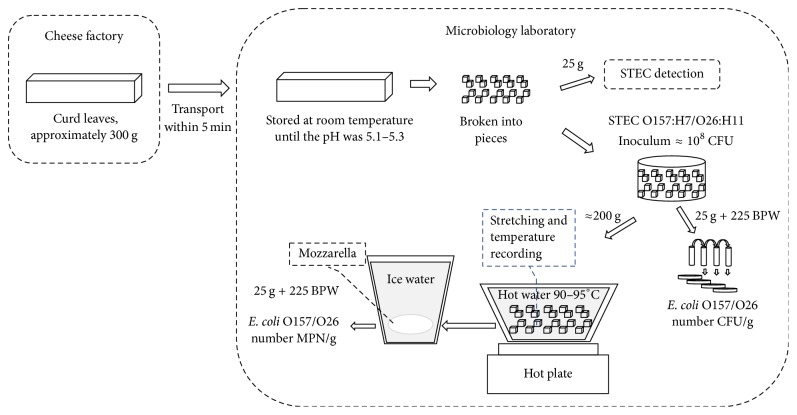
Schematic representation of the cheese processing conditions and microbiological analyses performed.

The scheme representing the complete process and analyses carried out is depicted in Figure 1.

### 2.6. Development of Nonisothermal Inactivation Models for Shiga Toxin-Producing* E. coli* O26 and O157 in Mozzarella Curd

For each serotype three nonisothermal temperature profiles and corresponding survival data of* E. coli* in the curd were integrated in the inactivation model described by Geeraerd et al. [22]. Regarding profiles tested for* E. coli* O26, the three profiles with the highest amount of data in the domain 64–80°C were selected for model development. The model is assuming that the inactivation process follows linear first-order kinetics with rate equal to* k*:

(1) $$\begin{array}{c} \frac{dN}{dt}=-k\cdot N. \end{array}$$

Subsequently, the parameter *k* is corrected by the addition of shoulder (*α*) and tail-shaped (*γ*) phases

(2) $$\begin{array}{c} k=-k_{max}\cdot \alpha \cdot \gamma , \end{array}$$

where *k*_max_ is defined as the maximum inactivation rate of the microorganism.

The tail-shaped curves were defined by an asymptotic function depending on *N*_res_:

(3) $$\begin{array}{c} \gamma =1-\left(\;\frac{N_{res}}{N}\;\right). \end{array}$$

The explicit solution of the dynamic model is defined in

(4) log⁡Nt=log⁡10logN0−10logNres·e−kmax·t+10logNres.

This model structure was chosen since a nonshoulder effect for* E. coli* inactivation in mozzarella curds was assumed, as previously obtained by Trevisani et al. [17] at isothermal conditions. As no shoulder effect was observed, the model was applied in its reduced version (log-linear tail) by omitting the shoulder parameter. Therefore, besides *k*_max_, the tailing effect was modelled by estimating the *N*_res_ parameter.

Initial contamination levels (*N*_0_, MPN/g) together with residual population (*N*_res_, MPN/g) were those measured at nonisothermal conditions.

Subsequently, relationship between kinetic parameters and treatment temperature (*T*) was performed through a secondary model equivalent to Bigelow's to describe the evolution of *k*_max_ with *T*:

(5) kmax=1AsymDT∗ln⁡10,

(6) log⁡AsymDT=log⁡AsymDref+Tref−Tz,

Asym*D*(*T*) (min) being the negative inverse slope of the log-linear part of the sigmoidal-like model at a given temperature [23, 24], Asym*D*_ref_ the Asym*D*-value at the reference temperature (*T*_ref_), and *z* the increase of temperature needed to produce a 90% reduction of the Asym*D*-value.

**Figure 2:**
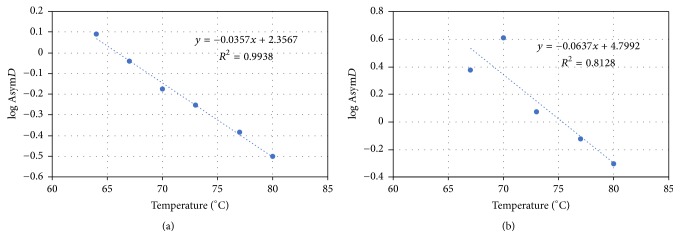
Linear regressions used to calculate *z* values (°C) representing evolution of log* AsymD* versus temperature. (a)* E. coli* O26; (b)* E. coli* O157.

For the present study, kinetic parameters obtained in the study of Trevisani et al. [17] were applied considering 70°C as the reference temperature (Table 1). Estimation of* z*-values was based on linear regressions of log* AsymD* against temperature for both STEC strains (Figure 2).

To solve the differential equations, the* Bioinactivation* package in *R* v3.2.3 was used [25].

The nonisothermal model estimated at each time-temperature profile was represented. Then, log reductions obtained in the current study at nonisothermal conditions (*N*_0_ – *N*_res_, log⁡MPN/g) were further compared with the estimated ones by the nonisothermal inactivation models. Statistical tests such as means comparison were made to find out significant differences (*P* < 0.05).

## 3. Results

### 3.1. Evolution of* E. coli* Serotypes during Curd Stretching of Mozzarella Cheese

All the curd loaves tested were negative for the presence of STEC. The use of defined starter cultures allowed reaching the pH values appropriate for stretching in 4 hours and the rheological characteristics of curd changed, making them ready to be moulded in shorter times (from 7.9–21.9 min to 1.5–5.1 min). The counts of O157 and O26 STEC before and after stretching, the times needed for stretching, and the temperature profiles in the curd are reported in Table 2. The maximum temperature values were in the range between 65 and 80°C. All tubes were positive for either O157 or O26* E. coli* by RT-PCR, but the presence of viable cells was confirmed only in some of them and these results were used to estimate the Most Probable Number (MPN). The water samples taken at the end of stretching had* E. coli* numbers below the lowest quantification level allowed by the MPN method, which for series of 10, 1, and 0.1 ml is equal to 0.03 MPN/ml (i.e., −1.52 log⁡MPN/ml).

### 3.2. Nonisothermal Modelling for the Inactivation of* E. coli* and Comparison with Observed Data

**Figure 3:**
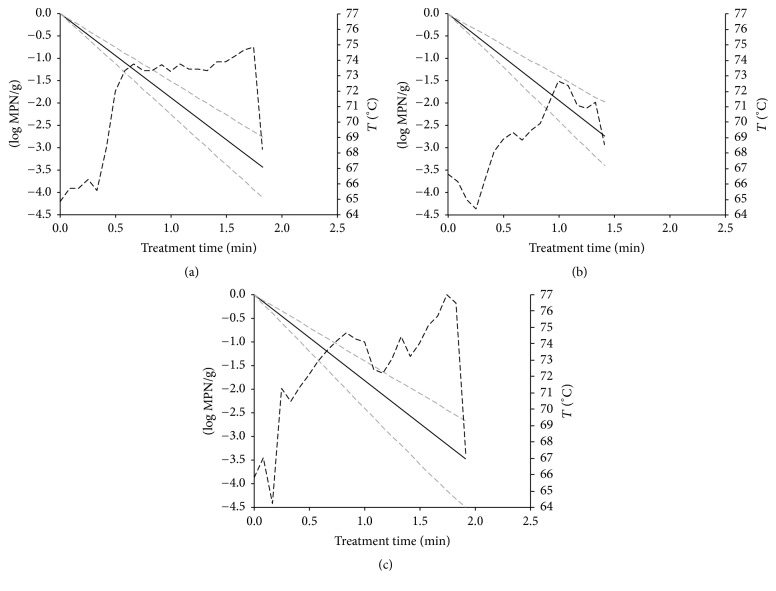
Estimated log reductions of* E. coli* O26 in mozzarella cheese at dynamic temperature conditions (temperature range: 64–80°C) during heat treatment of cheese curds. Continuous black line represents the mean log reductions while the grey dashed lines are the 95% CI. Dashed black lines describe the evolution of time/temperature conditions.

**Figure 4:**
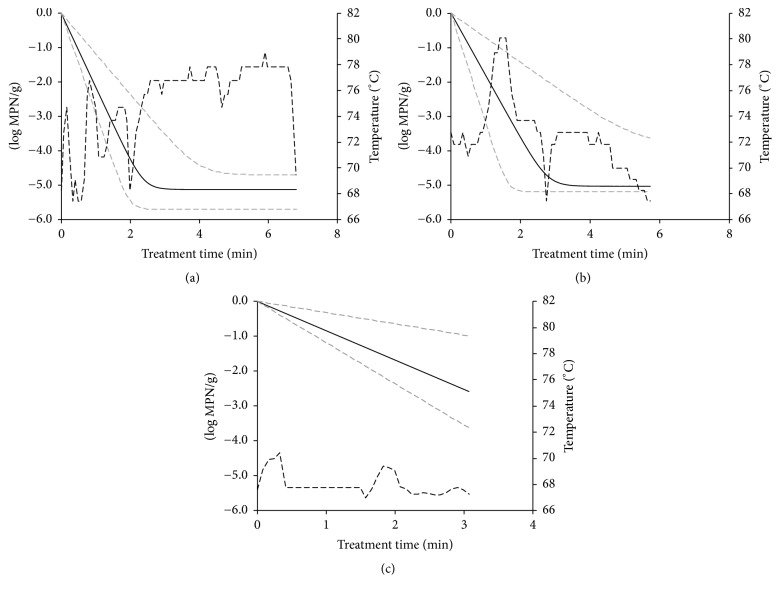
Estimated log reductions of* E. coli* O157 in mozzarella cheese at dynamic temperature conditions (temperature range: 67–80°C) during heat treatment of cheese curds. Continuous black line represents the mean log reductions while the grey dashed lines are the 95% CI. Dashed black lines describe the evolution of time/temperature conditions.

**Figure 5:**
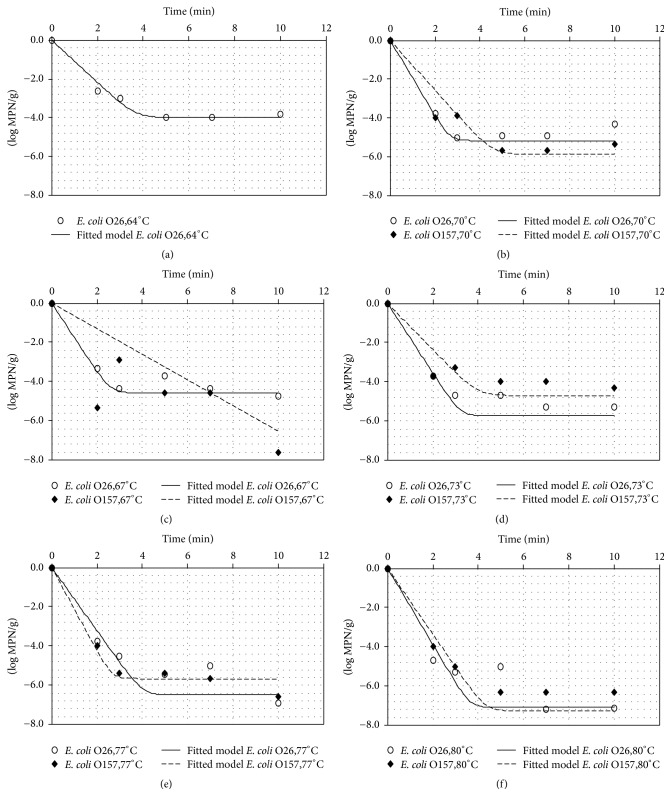
Comparison of the log-linear tail models at isothermal conditions in mozzarella cheese during temperature treatments of cheese curds for* E. coli* O26 at 64°C (a) and* E. coli* O26 and O157 at 67°C (b), 70°C (c), 73°C (d), 77°C (e), and 80°C (f). Observed data are available from Trevisani et al. (2014) together with additional data generated at 64°C for* E. coli* O26.

Predictions of log reductions over time were achieved using different *t*/*T* profiles within the studied temperature ranges (64–80°C for* E. coli* O26 and 67–80°C for* E. coli* O157). Estimated inactivation models of* E. coli* O157 and O26 at dynamic temperature conditions during temperature treatment of cheese curds are illustrated in Figures 3 and 4. Log reduction values were estimated as the difference between initial* E. coli* concentration and final population at the end of the treatment. These log reductions were calculated for the different temperature profiles and were then compared with the microbiological data observed (Table 3). The observed reduction of* E. coli* O157 and O26 during the thermal treatment varied between 3.55 (temperature profile 2 for* E. coli* O26) and more than 5.37 log⁡MPN/g (temperature profile 1 for* E. coli* O157), with lower reduction rates observed at the lowest temperatures and times (Table 3). It is shown that the comparison of predictions provided by the dynamic model with our own observations described well the linear inactivation pattern since nonsignificant differences were denoted at all profiles tested.  Figure 5 shows a comparison of the log-linear tail models in mozzarella cheese curds for* E. coli* O26 and O157 at isothermal conditions from the study of Trevisani et al. [17]. The log-linear tail-shaped curves fitted well at all temperatures treatments apart from the inactivation of* E. coli* O157 at 67°C, where a linear reduction was predicted.

## 4. Discussion

In this study, it was observed that stretching times are variable because they depend on the rheological characteristics of the curd. These differences were observed especially in the batches produced with the use of starters. Olivares et al. [26] studied the viscoelastic properties of mozzarella cheese and their dependence on the heating and ripening. Temperature of water during stretching depends on the water/curd mass and the addition of hot water/steam, whereas the core temperature depends on the time of treatment/curd mass ratio. The duration of the curd-stretching phase depends on the curd “stretchability,” which is affected by the rate of lactose fermentation and the starters inoculated in the raw milk [27]. Therefore, the use of pasteurization to inactivate pathogens should be required when the activity of starter cultures strongly reduces the duration of the stretching step.

In the five batches produced by using natural cultures (*cizza*) the time required to stretch the curd was longer and when the maximum core temperature reached maximum values above 78°C the number of* E. coli* (strain O157) was below the limit of quantification level allowed by the MPN method, which for tube series inoculated with 1, 0.1, and 0.01 g is equal to −0.52 log⁡MPN/g. Inactivation patterns shown in Figures 3 and 4 are attributed to the treatment times. Assuming microbial decay followed a linear trend, reductions did not present a tail at shorter times (2-3 min) or milder temperatures since inactivation was assumed to be linear. However, at longer treatments (>6 min) a tail-shaped curve was introduced in the model, since the existence of a residual subpopulation was assumed, which was proven in the experiments performed at static conditions. Considering the calculated 95% confidence intervals, there were no statistical differences (Table 3) between predicted and observed values. However, the observed mean log reduction values were often higher than those estimated by the model for* E. coli* O26. The differences were in the range of 0.99–1.51 log⁡MPN/g. These differences were not statistically significant due to the large confidence intervals of the MPN counts and of the model parameters *k*_max_ and *N*_res_.

The lower number of STEC observed in the curd was probably affected also by the dispersion of bacteria in the hot water, which was proven by the PCR analyses. Exponential amplification of target DNA sequences was observed at 31st–35th PCR cycles in the MPN tube series inoculated with 10 ml of water samples collected at the end of stretching. However, these water samples were negative using the culture detection method. Higher concentrations of the target DNA sequences (Ct values between 31 and 33) were detected in the batches that were produced using the natural starter that had longer stretching times (time-temperature profiles 1 and 2, Table 1). This factor was not considered in the experiments carried out at constant temperature and might have affected the estimates of the parameters *k*_max_ and *N*_res_. Potential deviations of the fitted regression models with the observed *k*_max_ and *N*_res_ values are mainly attributed to the nonlinear relationship of *k*_max_ and *N*_res_ with temperature. In the experiments at static temperatures this nonlinear dependency was affected by the rheological properties of curd that became very hard at mild-high heat treatments. This can reduce the movement of water and consequently the number of bacteria that were in an environment with low *a*_*w*_. There are previous studies that associate the presence of a residual population with a mechanistic approach (i.e., variability in the heating procedure of cell clumping) [21, 28]. This assumption is plausible with the food matrix tested in our study at static temperatures, where the reduced size of cheese curds favoured a heterogeneous distribution of cells, a protective effect of dead cells, and the acquisition of heat resistance mechanisms.

Although the public health risk related to the presence of STEC in raw milk is effectively reduced by the thermal treatments carried out during the stretching step, this also depends on the prevalence and concentration levels of STEC in raw milk. Only few studies reported data on the concentration of STEC in cow raw milk, which was relatively low (i.e., from less than 0.3 to 1.4 log⁡MPN/ml) [29, 30], whereas there is not any information regarding buffalo milk. The main reason might be the lack of an official quantitative technique and the difficulty in using the MPN method, which is laborious and time-consuming. Thus, the development of RT-PCR method for quantifying STEC in milk provides a less laborious and more convenient alternative [31].

The dynamic model developed in this study can be used to estimate the reduction of O157 and O26 STEC by measuring the temperature and time of the curd-stretching step within the studied inactivation ranges (64−80°C for* E. coli* O26 and 67−80°C for* E. coli* O157). Data concerning the growth of STEC during the processing step before stretching are lacking. Therefore, when assessing the thermal processing, it should be considered that STEC concentration in curd might be higher; thus, thermal processing should achieve a 5-log reduction of STEC.

Studies on the resistance of various strains of STEC and nonpathogenic* E. coli* to heat, acid, alkaline, and high hydrostatic pressure treatments found that variability in resistance was greater within strains of the same serotype than between strains of different serotypes. Moreover, variation in resistance was strain dependent rather than serotype dependent [32, 33]. The resistance of STEC isolates to temperature treatments could be related to specific protective mechanisms that are activated during the lag phase according to the medium composition [34]. The extent of external stresses (depletion of lactose and accumulation of organic acids) related to the use of starter cultures can significantly affect the resistance of STEC strains during the stretching phase.

According to the comparison between observed microbial reductions and those estimated by the developed models, it can be concluded that nonisothermal conditions can be appropriately reproduced. Therefore, it can be concluded that the predictive model can be useful in the field of food safety management since it allows the setting of Performance Objectives and Food Safety Objectives. Besides, how an operator achieves compliance can be specified [35]. For the cheese processing industry, the use of the predictive model could provide an application of performance and process/product criteria during the mozzarella cheese making process ensuring food safety of the final product.

## 5. Conclusions

The direct method of heat transfer that is used in the manufacture of mozzarella cheese is efficient for rapid heating and can easily inactivate the pathogenic microorganisms that are dispersed in the hot water, but the inactivation rates within the curd mass are affected by the kneading and pulling movements as well as other factors such as the duration of the curd-stretching step or the ratio between curd mass and hot water. A 5-log reduction in the concentration of O157 and O26 STEC can be achieved only with curd-stretching steps that bring the curd temperature at 78–80°C. The nonisothermal model developed in this study can be used to estimate the reduction of O157 and O26 STEC by measuring the temperature and time of the curd-stretching step within the studied inactivation ranges. Results obtained could be directly applicable by stakeholders since they provide a better understanding of microbial inactivation dynamics of STEC strains in mozzarella cheese production.

## Figures and Tables

**Table 1:** Kinetic parameters used in the dynamic model of Geeraerd et al. (2000) to predict inactivation of O157 and O26 *Escherichia coli*. Standard deviation values are represented between brackets.

Parameters	*E. coli *O26	*E. coli* O157
*T*_ref_ (°C)	70	70
*k*_max_ (min^−1^)^*∗*^	4.56 (0.57)	2.94 (1.21)
*AsymD*_ref_ (min)	0.505 (0.133)	0.783 (1.995)
log⁡*AsymD*_ref_	−0.296 (0.109)	−0.106 (0.503)
*z* (°C)	27.19 (5.563)	19.11 (2.418)
*N*_0_ (log⁡MPN/g)^*∗∗*^	4.45–4.85	4.39–4.53
*N*_res_ (log⁡MPN/g)^*∗∗*^	≤−0.52/0.63	−0.44/0.88

^*∗*^Averaged values of maximum inactivation rates were taken from Trevisani et al. (2014); ^*∗∗*^minimum and maximum values obtained before and after stretching.

**Table 2:** Characteristics of curd, temperature profiles recorded during the stretching phase, and changes in the counts (log⁡MPN/g) of O157 and O26 *Escherichia coli*.

	*E. coli* serogroup								
	O26						O157		
Microbial starters^*∗*^	N	N	N	A	A	A	N	N	A
Curd pH	5.1	5.0	5.2	5.0	5.1	5.1	5.2	5.0	5.3
Temperature profile	nc	nc	nc	1	2	3	1	2	3
Temperature range (°C)	Time in seconds								
56.1–64	325	102	147	21	22	6	23	144	109
64.1–66	650	204	294	21	22	6	16	69	68
66.1–68	11	90	27	14	18	6	17	69	102
68.1–70		40	1	12	13	4	24	75	39
70.1–72		4	1	4	27	9	32	88	10
72.1–74		2	1	7	24	16	40	134	
74.1–76				56	10	38	68	14	
76.1–78				19		36	221	9	
78.1–80						10	12	10	
>80								16	
Max temperature	65	71	64	75	73	77	79	80	72
Stretching time (min)	21.9	9.1	10.3	1.9	1.5	1.9	7.9	12.8	5.1
Count before stretching	4.23	4.12	4.05	4.39	4.43	4.53	4.85	4.46	4.45
Count after stretching	3.04	1.36	3.04	−0.04	0.88	0.44	<−0.53	<−0.53	0.63

^*∗*^N, natural whey left over from the previous day's *cizza* is added to lukewarm milk; A, artificial starter culture of *Streptococcus thermophilus* is inoculated in lukewarm milk; nc, not considered for the modelling purpose.

**Table 3:** Differences between the observed reduction of O157 and O26 Shiga toxin-producing *Escherichia coli* and the estimates produced on the basis of the inactivation kinetics of *E. coli* O26 and O157:H7 in mozzarella curds.

STEC serogroup	Temperature profile	Mean reduction (95% confidence interval)		Statistical significance
		Observed	Estimated	
O157	1	>5.37 (4.87–6.67)	4.36 (4.00–4.85)	*P* > 0.05
	2	>4.99 (4.48–6.29)	4.99 (4.24–5.16)	*P* > 0.05
	3	3.82 (3.20–4.50)	2.59 (0.94–3.47)	*P* > 0.05
O26	1	4.43 (3.81–5.24)	3.68 (2.95–4.41)	*P* > 0.05
	2	3.55 (3.13–4.20)	2.68 (2.04–3.42)	*P* > 0.05
	3	4.98 (4.28–6.30)	3.97 (3.02–5.07)	*P* > 0.05

Values calculated from the difference between the counts in curd (log⁡CFU/g) and in “*pasta filata*” (log⁡MPN/g). Differences with symbol > indicate MPN counts below the limit of quantification at the end of stretching.
